# Recurrent Network Solutions for Human Posture Recognition Based on Kinect Skeletal Data

**DOI:** 10.3390/s23115260

**Published:** 2023-06-01

**Authors:** Bruna Maria Vittoria Guerra, Stefano Ramat, Giorgio Beltrami, Micaela Schmid

**Affiliations:** Laboratory of Bioengineering, Department of Electrical, Computer and Biomedical Engineering, University of Pavia, 27100 Pavia, Italy; brunamariavitt.guerra01@universitadipavia.it (B.M.V.G.); giorgio.beltrami@unipv.it (G.B.); micaela.schmid@unipv.it (M.S.)

**Keywords:** human action recognition, ambient assisted living, deep learning, recurrent neural network, skeletal data

## Abstract

Ambient Assisted Living (AAL) systems are designed to provide unobtrusive and user-friendly support in daily life and can be used for monitoring frail people based on various types of sensors, including wearables and cameras. Although cameras can be perceived as intrusive in terms of privacy, low-cost RGB-D devices (i.e., Kinect V2) that extract skeletal data can partially overcome these limits. In addition, deep learning-based algorithms, such as Recurrent Neural Networks (RNNs), can be trained on skeletal tracking data to automatically identify different human postures in the AAL domain. In this study, we investigate the performance of two RNN models (2BLSTM and 3BGRU) in identifying daily living postures and potentially dangerous situations in a home monitoring system, based on 3D skeletal data acquired with Kinect V2. We tested the RNN models with two different feature sets: one consisting of eight human-crafted kinematic features selected by a genetic algorithm, and another consisting of 52 ego-centric 3D coordinates of each considered skeleton joint, plus the subject’s distance from the Kinect V2. To improve the generalization ability of the 3BGRU model, we also applied a data augmentation method to balance the training dataset. With this last solution we reached an accuracy of 88%, the best we achieved so far.

## 1. Introduction

The world is facing a significant demographic change: the aging population is increasing at an unprecedented rate in all countries. It is estimated that the elderly world population over 60 years of age will increase to 2.1 billion people by 2050, compared to 1 billion estimated in 2020 [1,2]. Such an increase of the global aging population is associated with age-related challenges, such as reduced mobility, falls, difficulties in performing daily activities, memory-related and social isolation issues, which have led the society and the different national health care systems to face ever-growing demand for monitoring, assistance, and medical care. Moreover, the recent COVID-19 pandemic has stressed this situation even further, thus highlighting the need for taking action [3,4].

Ambient Assisted Living (AAL) technologies come as a viable approach to meet these challenges, thanks to the high potential they have in enabling remote care and support [5]. AAL systems are designed to provide support in daily life in an unobtrusive and user-friendly manner. Moreover, they are conceived to be smart, to be able to learn and adapt to the requirements and the requests of the assisted people, and to join with their specific needs. One of their possible applications regards the monitoring of the elderly based on different types of sensors, including wearables, environmental and cameras, to collect a large amount of information ranging from habits to vital parameters of the inhabitant [6]. For this purpose, wearable devices are largely used for the numerous advantages they offer, including their small size, the low energy demand necessary for their operation, and the full respect for the subject’s privacy [7]. Nevertheless, they have also some drawbacks. For example they need to be worn by the subjects and to be frequently recharged. These issues could be a significant problem for the elderly subject. Moreover, to fully capture the 3D motion associated with a human action, a single sensor may not be adequate. It may be necessary to utilize multiple sensors, thus increasing the intrusiveness of the devices worn by the subject [8,9,10]. In this context, cameras are recommended, since they overcome all these limits. Usually, the architecture of the AAL vision-based solutions consists of a single camera or a set of cameras, installed in the home environment, capturing the data that is later analyzed by processing and decision modules assessing the opportunity to produce an alarm for third parties (i.e., caregiver, human operator, ambulance and so on). Indeed, cameras are far less obtrusive with respect to the burden other wearable sensors may impose on one’s activities [5,11,12]. Nevertheless, cameras are often perceived as one of the most intrusive technologies in terms of the privacy of the monitored individuals. The solution to this drawback may be low-cost RGB-D cameras, which offer the possibility to extract the “skeleton” of the subject from the depth image, depicting the subject as a set of body segments and joints. The use of skeletal tracking for monitoring purposes increases the person’s acceptance of the camera, since it partially preserves privacy [11]. In the AAL domain, these skeleton data can be used to automatically identify different human activities by means of a plethora of Artificial Intelligence (AI) algorithms characteristic of the Human Activity Recognition (HAR) processes [13]. Due to the recent advancements in computing power, deep learning-based algorithms have become the most effective and efficient choice for recognizing and solving HAR problems. In this context, deep learning solutions are trained on the data collected from a sensor, or a set of sensors, in order to automatically identify the user’s activities [14]. The most attractive deep learning architectures for skeleton-based HAR are Recurrent Neural Networks (RNN) [4,15,16,17,18,19,20,21,22].

RNNs have the capability to label data sequences or time series; they are able to keep the ‘memory’ from previous input sequences, which in turn influences the output of the current sequence. Therefore, unlike traditional machine learning algorithms, from K-nearest neighbor to multi-layer perceptron, RNNs do not assume that the data sequences are independent from each other, so that the information learnt from the prior sequences is used to learn from the current sequence. RNNs introduce the concept of “state” of the network, as they become a dynamical system for which the output depends on its history. For this reason, RNNs are commonly used to find out the dynamics of the data time series by taking advantage of their temporal structures.. Long Short-Term Memory (LSTM) [23] and Gated Recurrent Units (GRUs) [24] are two different kinds of RNN architectures, both characterized by internal mechanisms (four for the LSTM and two for the GRU), called gates, able to regulate the flow of information. These gates can discern, in a sequence of data, those more significant for the classification, and hence to be kept in the process, from those less meaningful, to be excluded. This allows the network to select the relevant information from all the data sequences, keeping the ‘memory’ not only of the previous sequence but of all learned data [15,25,26,27].

In this study, we investigate the performance of different RNN models to classify time series of skeleton-tracking data in order to identify both some daily living and unconventional postures assumed by a person in a room. This classification process is the core of a more complex home monitoring system designed by our group, and still in its tuning phase, which is conceived to recognize dangerous situations or voluntary requests for help. Briefly, the system, which is tailored to frail people living alone, consists of three main blocks working in series: acquisition, classification and decision block. The first block manages the data acquisition through a network of four Kinect V2 (Microsoft, Redmond, WA, USA), the data pre-processing and the transmission toward the classification block. This latter, based on a deep learning model, classifies the input data in terms of postures. The posture identified, one for each Kinect V2 data, paired with its classification accuracy, is finally sent to the last block that is responsible for making a decision. At this level the most reliably identified body posture is selected and integrated with the position of the person in the room and with respect to that of the furniture. This last process allows us to distinguish a scenario of daily life from a potentially dangerous situation (for example, a person lying in bed → probable everyday life situation; person lying on the ground → potentially dangerous scenario) in order to produce an alarm for a third person only if appropriate.

Different feature selection methods, as well as different machine and deep learning architectures, were proposed for the classification block in previous studies by our group [28,29]. The most promising solution was proposed in Guerra et al. [30], where a genetic algorithm was applied to select eight kinematic features and a sequence-to-sequence model was trained to identify five classes. Three classes correspond to the three postures frequently adopted by a person during daily activities: standing, sitting, and lying down; one represents an unconventional daily posture, labeled “dangerous-sitting”, and groups all postures that in some way manifest a malaise or fainting, such as a seated person slumped or lying backward; and the last class groups all the transitions between two consecutive postures (for example, between sitting and lying postures and vice-versa). The dangerous-sitting class was defined in order to allow, at the level of the classification block, an initial distinction between routine activities and alarm situations. Therefore, for the efficiency of the home monitoring system, the specificity of the classifying model for such a class is extremely important for reducing the number of false negatives. The model, characterized by two Bidirectional Long Short-Term Memory layers, alternated by two dropout layers and, as last layer, a fully connected layer (2BLSTM2D), reached an overall accuracy of 85.7% and a percentage of about 85% and 95% regarding the specificity and sensitivity metrics of the dangerous-sitting posture [30]. Here, aiming to take advantage of the temporal dependency of the inputs to further improve the accuracy of the classification block and, in particular, its specificity for the dangerous-sitting class, a new deep RNN architecture based on GRU networks with a sequence-to-last approach was trained and tested to identify the five classes described above. We hypothesized that a GRU model, having fewer parameters than the LSTM one given the same number of units, could learn better with our somewhat limited dataset size. Our GRU-based model was characterized by three Bidirectional GRU layers, alternated by four dropout layers, and three fully connected layers (3BGRU), for a total of about 220 k hyperparameters (the 2BLSTM2D model was described with roughly 460 k).

At first, the features considered were those previously selected by the genetic algorithm [30,31,32]. As a second step, inspired by the work of Wang et al. [33], we tested the hypothesis that the performance of this new architecture could increase by using a large number of raw kinematic features (i.e., joints coordinates) instead of the reduced set of structured features selected with the genetic algorithm (eight kinematic features: articular angles, absolute angles and vertical joint positions [30]). To this end, we defined a new set of 52 features based on 3D skeleton joint coordinates, computed in an egocentric reference system, and the Euclidean distance of the subject from the Kinect V2 which was acquiring the data [34]. Finally, as a last step, focusing on the idea that the home monitoring system must recognize the dangerous situation immediately as its occurs rather than during it, we applied the same architecture to classify only four classes, abolishing transition one (previous Class 5). Specifically, the data referring to the transition between two consecutive postures have been labelled with the class corresponding to the posture following the transition. For example, if the subject changes from a standing to sitting posture, the data referring to the transition are labelled as the sitting posture class. The performance of this latter architecture was compared with the one previously proposed [30], yet with a sequence-to-last approach, 52 input features and four output classes, which will be referred to as the 2BLSTM2D model. Finally, to increase the generalization abilities of the 3BGRU model we also applied a data augmentation method for rebalancing the training dataset [35].

## 2. Materials and Methods

The subjects involved in the study as well as the experimental set-up employed to acquire the data have been the same detailed in [28]. Therefore, hereinafter only a brief overview of the fundamental information is provided.

### 2.1. Subjects

Twelve subjects (seven females and five males; age ranging 25 and 60 years old) participated in the study. All subjects gave written informed consent in accordance with the Declaration of Helsinki.

### 2.2. Experimental Set-Up

The data were acquired with four Kinect V2 devices, arranged in a prototyped room according to a configuration that allows covering the largest possible room area: two of them were positioned to see the whole room, while the remaining were placed to specifically acquire two areas of the room, i.e., the bed and the desk. The four Kinect V2 recorded the scene simultaneously but the captured data were processed separately. A custom-made C#-based tool with GUI was developed using VisualStudio 2017 to control the Kinect V2 acquisitions.

**Figure 1:**
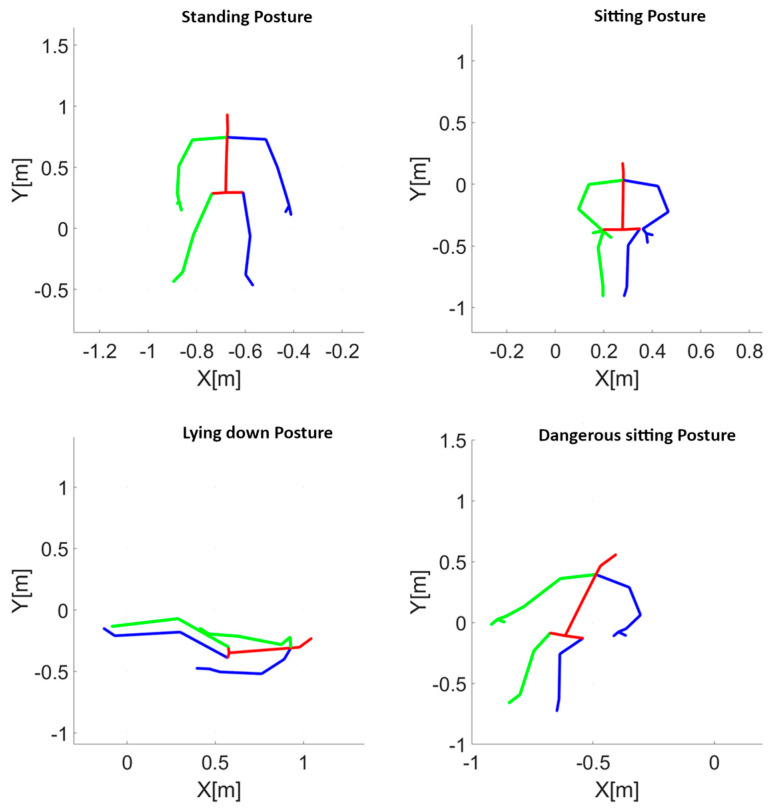
Example of the four postures acquired with the Kinect V2 camera. Standing posture (**top left** panel), sitting posture (**top right** panel), lying posture (**bottom left** panel) and dangerous–sitting posture consisting in slumping on a chair with the head leaned backward (**bottom right** panel) are depicted in the Kinect V2 spatial reference system. In the visualization, the red lines indicate the body segments of the head, trunk, and pelvis. The green lines represent the body segments of the right hemi-body, including the shoulder, arm, forearm, hand, thigh, leg, and foot. Similarly, the blue lines represent the body segments of the left hemi-body.

A total of 265 trials of about 13 min each were recorded. In each trial, subjects were asked to adopt an ordered sequence of postures (standing, sitting, lying, and slumping in a chair with the head leaned forward or backward). An exemplification, in the form of body stick diagrams, of the four different postures required of the subjects is depicted in the panels of Figure 1. Each posture was taken for about 10 s, whereas the transitioning from one posture to the following one lasted about 1 s.

### 2.3. Data Analysis

**Figure 2:**
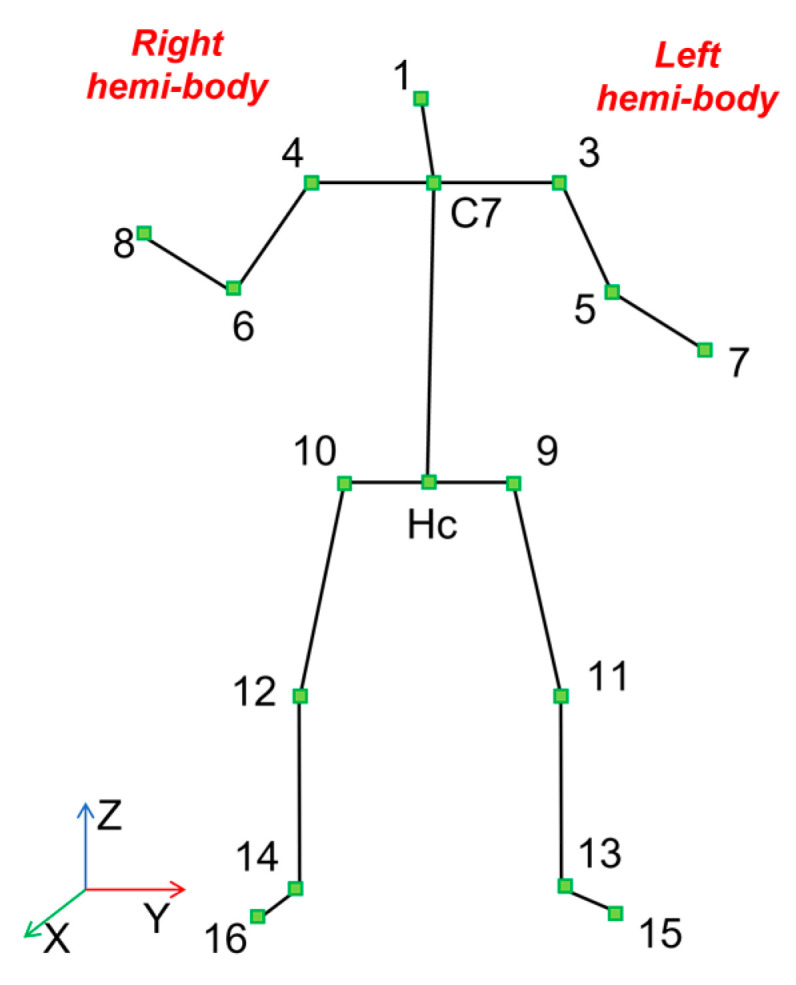
The 17 joints skeleton considered from each Kinect V2 recording [30] Each number corresponds to a specific point of repere of the body used to reconstruct the skeletal stick diagram.

From the data of each Kinect V2, the spatial coordinates (x, y, z) of 17 skeletal joints (Figure 2) were estimated by a custom-developed software based on the Kinect’s SDK. In order to make comparable the data of the four Kinects, all joint coordinates were roto-translated to be referred to a global reference system (*X*, *Y*, *Z*). The set of kinematic features previously defined in Guerra et al. [28] (articular angles, head and trunk pitch and roll angles and head, C7 (mid-point between the shoulder joints) and Hc (mid-point between the hip joints) vertical position) were computed. Taking into account the strong correlation between the accuracy of the acquisition data and the position of the subject with respect to the camera, we considered another regressor: the Euclidean distance between the C7 joint and the Kinect V2 position (see Equation (1)).

(1) $$D\left(C7,\;K_{n}\right)=\sqrt{{\left(X_{C7}-X_{K_{n}}\right)}^{2}+{\left(Y_{C7}-Y_{K_{n}}\right)}^{2}+{\left(Z_{C7}-Z_{K_{n}}\right)}^{2}}$$

where n=1,…4 (number of Kinect V2 system).

Finally, the 3D skeleton joint coordinates were also computed in an egocentric reference system centered in the C7 joint [36,37,38,39,40,41]. All feature values were normalized: the angle values were divided by 180; the head, C7 and Hc vertical absolute position and all the ego-centric joint coordinates were scaled with respect to the subject’s height; the $D\left(C7,\;K_{n}\right)$ were computed between 0 and the depth value of the room (5 m).

In order to maintain the temporal consistency among the data of each Kinect V2 system, the frames referring to the missing data, principally due to the transient exit of the subject from the camera view or when the subject was not facing the camera, have been filled with the value of 999. Finally, a moving mean filter, with a 15-frame (equal to 0.5 s) time window, was carried out. If the time window contained only 999 values (missing data), the mean was not calculated, and the 999 value was retained.

### 2.4. Dataset Construction

Four datasets were created. In each dataset, the training data consisted of data collected from 10 out of 12 subjects, while the test data included data from the remaining two subjects. To maintain inter-subject variability in the test set, a tall male and a short female subject were selected, with the female subject being younger than the male. The datasets were divided into temporal sequences of 120 frames, i.e., four seconds of data. For the training data an overlapping of 60 frames (50%) was considered. Each frame in a temporal sequence was labeled with the class corresponding to the majority class in the sequence.

The *first dataset* was characterized by eight features (pitch and roll angles of the head and trunk, angle between head and shoulder segments, angle between trunk and hip segments, vertical position in the global reference system of the C7 and Hc joints (see Guerra et al.: $\;A_{pitch}$, $A_{roll}$, $B_{pitch}$, $B_{roll},\;µ2,\;\delta 2$, $Z_{C7}$, $Z_{Hc}$) [30]) and five classes (Class 1: standing posture; Class 2: sitting posture; Class 3: lying posture; Class 4: dangerous-sitting posture; Class 5: transition between two consecutive postures). The full database included a total of 9124 sequences including those containing the 999 values. The class subdivision of the collected data is shown in Table 1.

The *second dataset* was characterized by 52 features (Euclidean distance between C7 joint and the Kinect V2 position, and the egocentric coordinates of all 17 joints) and five classes (Class 1: standing posture; Class 2: sitting posture; Class 3: lying posture; Class 4: dangerous-sitting posture; Class 5: transition between two consecutive postures). The full database included a total of 9500 sequences including those containing the 999 values. The class subdivision of the collected data is shown in Table 1.

The *third dataset* was characterized by 52 features (Euclidean distance between C7 joint and the Kinect V2 position and the ego-centric coordinates of all joints) and four classes (Class 1: standing posture; Class 2: sitting posture; Class 3: lying posture; Class 4: dangerous-sitting posture). In this case, the frames referring to the transition between two consecutive postures were labeled with the class of the posture reached at the end of the transition. For example, if the subject changes from a standing to sitting posture, the data referring to the transition are labelled with the sitting posture class; vice versa, if the person changes from a sitting to a standing posture, the data referring to the transition are labelled with the standing posture class. The class repartition of the new dataset is shown in Table 2.

**Figure 3:**
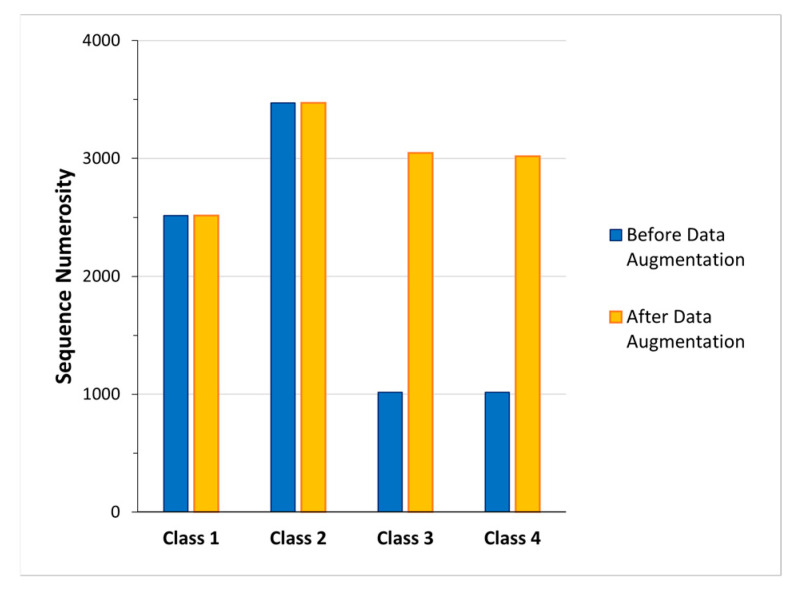
Numerosity of the sequences before (blue bars) and after (yellow bars) the data augmentation process adopted to increase the cardinality of Class 3 and 4.

Finally, the *fourth dataset* was characterized by 52 features (Euclidean distance between C7 joint and the Kinect V2 position, and the ego-centric coordinates of all joints) and four classes (Class 1: standing posture; Class 2: sitting posture; Class 3: lying posture; Class 4: dangerous-sitting posture). In this dataset, a data augmentation procedure was carried out for Class 3 and Class 4, those with fewer examples (Figure 3) [42]. The data augmentation was performed by adding to the data of each sequence a Gaussian noise (calculated on the mean and standard deviation of the data belonging to the sequence) [43,44]. The class splitting of the new dataset is shown in Figure 3 and the reported partitions of training and test conditions are shown in Table 2.

### 2.5. Deep Learning Architecture

**Figure 4:**
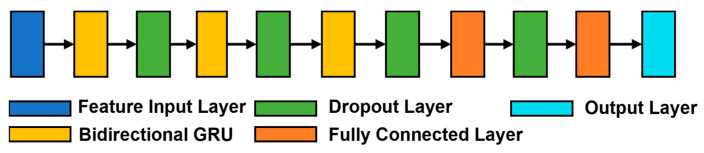
The architecture of the 3BGRU model.

The new model that we developed to classify the different datasets was based on the Bidirectional GRU model. A sketch of the architecture summarizing the different layers is shown in Figure 4. The model (3BGRU) was composed of a first feature input layer (blue block in the figure), characterized by a masking property allowing the model to ignore the 999 samples while maintaining the temporal sequence of the data, followed by a GRU layer with 100 hidden neurons (yellow block in the figure) and a dropout layer (green block in the figure), for preventing overfitting, with a dropout percentage of 40%. This sequence of layers was then repeated twice, yet with 50 hidden neurons in the GRU layer, and was then followed by a fully connected layer (orange block in the figure) with 50 hidden neurons and a new 40% dropout layer. The architecture ended with a fully connected layer with 25 hidden neurons, and an output layer (cyan block in the figure) implemented with a softmax activation function. The model was configured for sequence-to-last classification.

To obtain a statistical assessment of its performance, the model was trained and tested for 30 simulations on each considered dataset.

### 2.6. Statistical Analysis

For each dataset and for each simulation we computed the accuracy, precision, sensitivity and specificity. For model simulations on the third and fourth datasets, the classification error was also calculated. The latter was defined, for each class, as the ratio between the number of classification errors and the number of sequences labeled as belonging to such class (False Negative Rate, FNR; the formula is reported in Equation (2)). For the 3BGRU architecture tested on each of the four datasets, and for the 2BLSTM one tested on the *third dataset* (52 features and four classes), the mean value and the standard deviation of each considered metric were computed over the 30 simulations. Moreover, for the 3BGRU architecture as well as for the 2BLSTM architecture, the mean confusion matrix was calculated as the mean of the 30 confusion matrices, and all the cells of the mean matrix were normalized with respect to the frame cardinality of each class.

The ROC curve was also computed for each of the 30 network simulations, and the mean ROC curve was then obtained by averaging them.

Finally, to explore the performance of the different proposed solutions, the mean accuracy results of the two architectures classifying five classes were compared with a *t*-test, whereas those of the three architectures classifying four classes were compared using a one-way ANOVA test. For both statistical tests, the alpha level was set at 0.05. Considering the overall goal of the proposed system, i.e., monitoring the frail individual to raise an alarm in case a dangerous situation is detected, we focused our analysis on the performance of the different proposed solutions relative to the identification of Class 4 (dangerous-sitting class). Accordingly, the comparisons were made in terms of specificity and sensitivity of Class 4, applying the same statistical tests adopted for the mean accuracy results.

(2) $$FNR=\frac{FN}{FN+TP}$$

## 3. Results

### 3.1. First Dataset (Eight Features—Five Classes)—3BGRU Architecture

During the training, the model received 8511 input sequences (see Table 1), each one composed of 120 frames, with a 50% overlap. The sequences were labelled based on the majority class for its constituent frames among the five classes considered (sequence-to-last model). The validation dataset corresponded to the 10% of the training data. The test sequences were, instead, 1176 in total.

The mean accuracy value of the model was equal to 0.82 ± 0.01.

Overall, the 3BGRU architecture applied to the first dataset (eight features—five classes) achieved high mean specificity values (ranging from 0.92 ± 0.01 to 0.97 ± 0.01; see Table 3) across all classes, indicating a low rate of False Positives (FP). The lowest mean value was that of Class 2 (Table 3). The sensitivity mean values varied widely across classes, ranging from 0.62 ± 0.05 for Class 5 to 0.96 ± 0.01 for Class 3 (see Table 3), suggesting that the model’s ability to identify True Positives (TP) differed depending on the class. In terms of precision, the architecture achieved mean values ranging from 0.73 ± 0.03 for Class 5 to 0.87 ± 0.03 for Class 1 (see Table 3), indicating that the model may produce more FP for some classes than others. Notably, the SDs for these measures were relatively small (ranging from 0.01 to 0.05), implying that the results were consistent across the 30 simulations.

**Figure 5:**
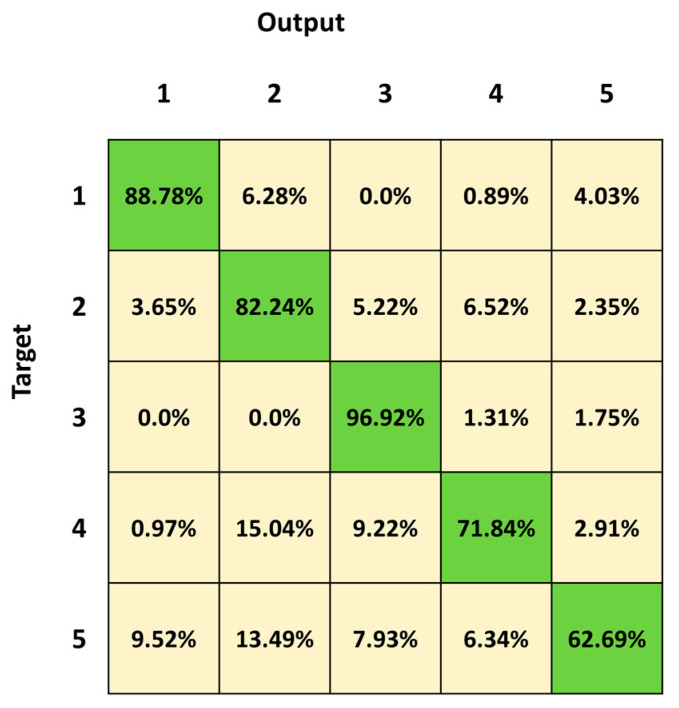
Mean confusion matrix obtained over 30 3BGRU architecture simulations with the *first dataset*.

Figure 5 depicts the mean confusion matrix computed over the results of the 30 network simulations. It summarizes the average values of the FP, False Negatives (FN), True Negatives (TN) and TP for each class. The most important misclassifications involve Class 4 and Class 5, with a percentage of correct classifications of 71.84% and 62.69%, respectively. Both classes are mainly confused with Class 2 (15.04% and 13.49% for Class 4 and 5, respectively). Class 5 is also confused with all the other three classes (9.52%, 7.93% and 6.94%, respectively Class 1, Class 3 and Class 4). The best identified class is Class 3 (lying posture), followed by Class 1 (standing posture).

### 3.2. Second Dataset (52 Features—Five Classes)—3BGRU Architecture

During the training, the model received 8511 input sequences, each one composed of 120 frames, with a 50% overlap. The sequences were labelled based on the majority class for its constituent frames among the four classes considered (sequence-to-last model).

The validation dataset corresponded to the 10% of the training data. The test sequences were 989 in total.

The mean accuracy value of the model was equal to 0.81 ± 0.01.

As summarized in Table 3, the 3BGRU architecture applied to the *second dataset* (52 features—five classes) achieved high mean specificity values ranging from 0.89 ± 0.01 to 0.99 ± 0.01, indicating a low rate of FP. The highest mean specificity result was that of Class 5 at the expense of a very low mean value of sensitivity (0.19 ± 0.07), indicating that the model had difficulty in correctly identifying TP. The precision mean values ranged from 0.47 ± 0.09 for Class 5 to 0.88 ± 0.02 for Class 3, meaning that the model may produce more FP in classifying Class 5.

**Figure 6:**
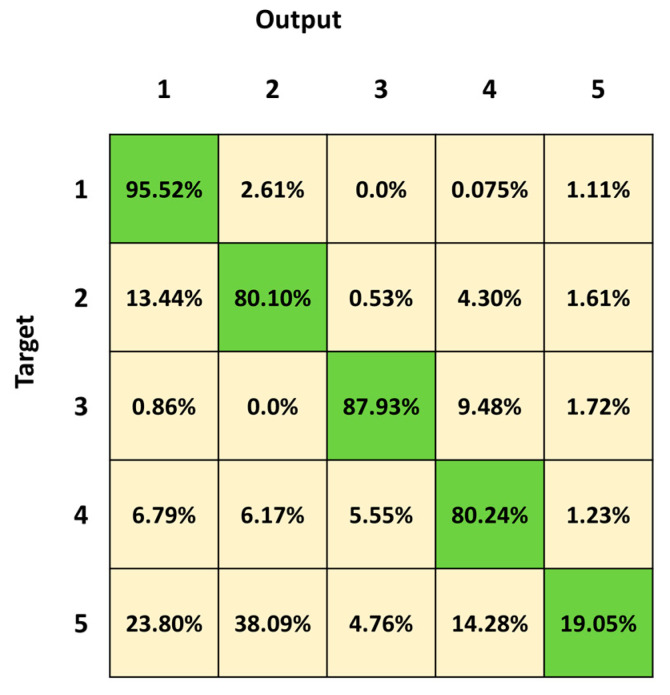
Mean confusion matrix obtained by 30 3BGRU architecture simulations with the *second dataset*.

Figure 6 shows the mean confusion matrix computed over the results of the 30 network simulations. Observing the classification results, the most important misclassifications are in Class 5, followed by Class 4 and Class 2, with a percentage of correct classifications of 19.05%, 80.24% and 80.10%, respectively. The classifier frequently fails in the identification of Class 5, which is confused with all the other four classes (23.80%, 38.09%, 4.76% and 14.28%, respectively). Class 2 is mainly confused with Class 1 (13.44%) and Class 4 (4.30%). The best identified class is Class 1 (standing posture), followed by Class 3 (lying posture).

### 3.3. Third Dataset (52 Features—Four Classes)—3BGRU Architecture

The model was trained over 8511 input sequences, each one composed of 120 frames, with a 50% overlap. The sequences were labelled based on the majority class for its constituent frames among the four classes considered (sequence-to-last model).

The validation dataset corresponded to the 10% of the training data. The test sequences were 989 in total.

The mean accuracy value of the model was equal to 0.87 ± 0.01.

The model achieved high mean specificity values for all classes, ranging from 0.93 ± 0.02 to 0.98 ± 0.01, indicating a low rate of FP (see Table 4). The mean sensitivity values were also relatively high for Classes 1, 2, and 3, ranging from 0.83 ± 0.04 to 0.91 ± 0.03, indicating a low rate of FN (see Table 4). However, for Class 4, the mean sensitivity value was relatively low (0.83 ± 0.04), suggesting that the model had difficulty correctly identifying the TP of this class (see Table 4). The mean precision values ranged from 0.81 ± 0.02 for Class 4 to 0.90 ± 0.02 for Class 2, implying that the model may produce more FP for Class 4 and may have difficulty distinguishing between TP and FP for some classes (see Table 4).

**Figure 7:**
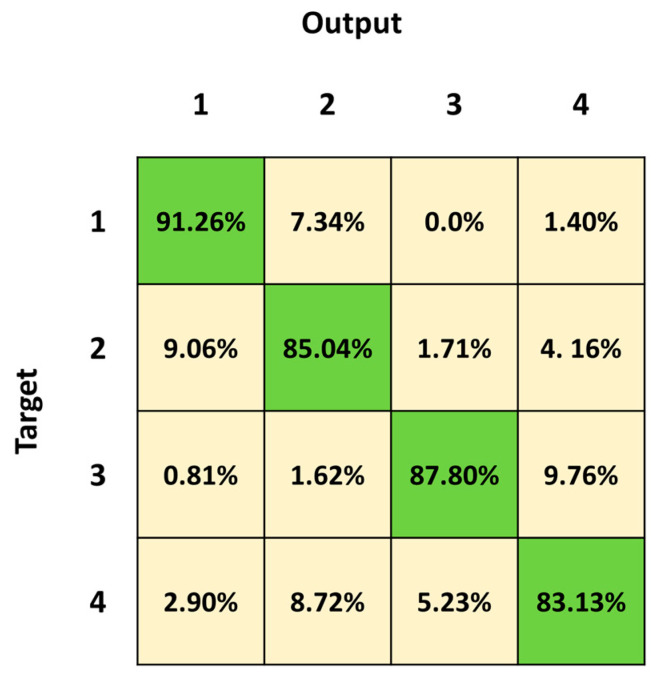
Mean confusion matrix obtained over 30 3BGRU architecture simulations with the *third dataset*.

Figure 7 shows the mean confusion matrix computed over the results of the 30 network simulations. The major misclassifications are in Class 4, Class 2 followed by Class 3, with a percentage of correct classification of 83.13%, 85.04% and 87.80%, respectively. Class 2 is mainly confused with Class 1 (9.06%) and vice versa (7.34%), whereas Class 2 is mistaken with Class 4 (4.16%) and vice versa (8.72%). Class 4 is also misclassified with Class 3 (5.25%) and vice versa (9.76%). The best identified class is Class 1 (standing posture), followed by Class 3 (lying posture) and Class 2 (sitting posture).

**Figure 8:**
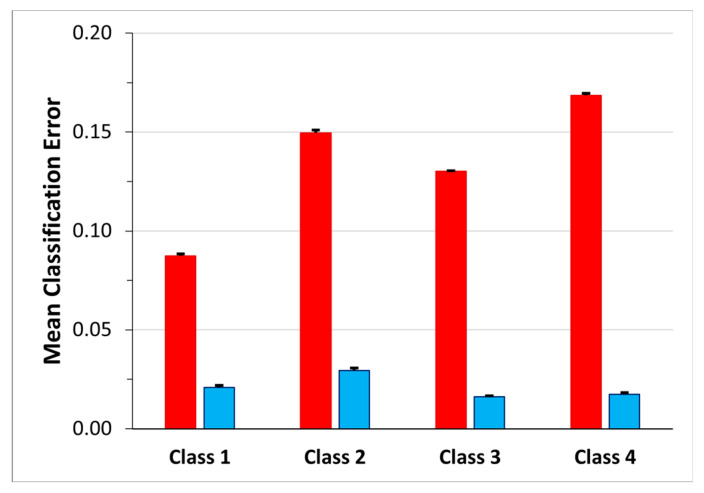
Mean classification error for each class. The red bars represent the percentage of FN for each class, while the blue bars represent the percentage of FN that corresponds to frames that were previously considered transitions and now are labeled as pertaining to the class.

In order to gain a better understanding of how the network manages the transition frames and to investigate the effectiveness of the choice of identifying four classes instead of five, considering the transition frames as belonging to one of these four classes and not to a dedicated class, we analyzed the classification errors. Moreover, for each class, to understand how much the transitions affected the classification error we computed the ratio between the number of the transition frames labelled with the class and the numerosity of the classification error (transition frames error ratio). The mean value over the 30 network simulations is shown, for each class separately, in the blue bar of Figure 8. The highest mean classification error occurs in Class 4 (0.17 ± 0.14) followed by Class 2 (0.15 ± 0.09). Class 2 even shows the highest mean transition frames error ratio (0.03 ± 0.12), whereas the lowest one is that of Class 3 (0.016 ± 0.04).

### 3.4. Third Dataset (52 Features—Four Classes) and 2BLSTM Architecture

The 2BLSTM architecture, defined in Guerra et al. [30], was adapted to run with the third dataset with a sequence-to-last classification approach, as used for the training of the 3BGRU model. Thus, during the training the model received as input 8511 sequences composed of 120 frames each and described by the 52 features. Again, the validation dataset consisted of the 10% of the training data and the test sequences were 989 in total.

The mean accuracy value of the model was equal to 0. 85 ± 0.01.

The results summarized in Table 4 show high mean specificity values for all classes, ranging from 0.92 ± 0.02 to 0.98 ± 0.01, indicating a low rate of FP. The mean sensitivity values of Class 2 and Class 3 were slightly lower compared to the other two classes (0.80 ± 0.03 and 0.80 ± 0.07), implying that the model had more difficulty correctly identifying TP for these classes. Conversely, the mean sensitivity values of the other two classes were high, 0.84 ±0.05 for Class 4 and 0.93 ± 0.02 for Class 1, suggesting that the model was capable of accurately identifying TP for these classes. Furthermore, the mean precision values ranged from 0.79 ± 0.03 for Class 4 to 0.89 ± 0.04 for Class 2, indicating that the model had more difficulty in distinguishing between TP and FP for Class 4.

**Figure 9:**
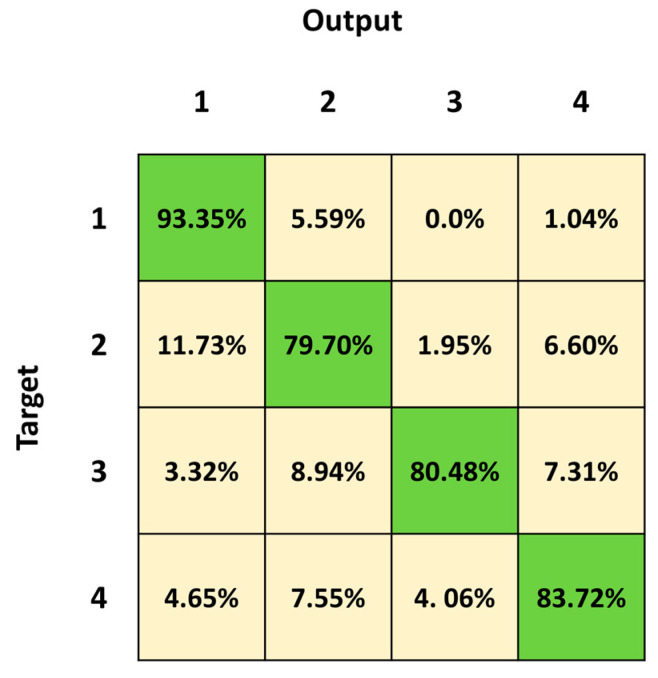
Mean confusion matrix obtained by 30 2BLSTM architecture simulations with the *third dataset*.

**Figure 10:**
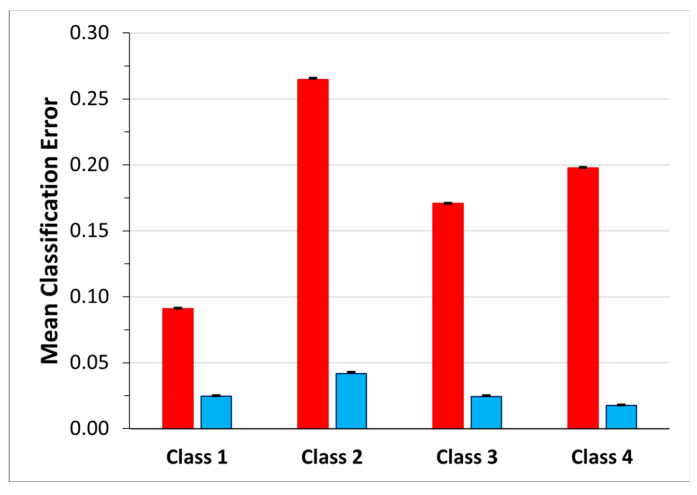
Mean classification error for each class for the 3BGRU model on the *third dataset*. The red bars represent the percentage of FN for each class, while the blue bars represent the percentage of FN that corresponds to frames that were previously considered transitions and now are labeled as pertaining to the class.

Figure 9 shows the mean confusion matrix computed over the results of the 30 network simulations. The highest values of misclassifications are in Class 2 and Class 3 followed by Class 4, with a percentage of correct classification of 79.70%, 80.48% and 83.79%, respectively. Class 2 sequences were mainly confused with Class 1 (11.73%) and Class 4 (6.60%). The best identified class was Class 1 (standing posture), followed by Class 4 (dangerous-sitting posture). The mean classification errors are also depicted as red bars in Figure 10. Note that the highest mean classification error occurs in Class 2 (0.20 ± 0.12), which also showed the highest ratio of false negatives that had previously been considered as transitions (0.03 ± 0.08).

As previously described (see *Third dataset* (52 features—four classes)—3BGRU architecture paragraph) the mean classification error and the mean transition frames error ratio were computed and shown in Figure 10.

### 3.5. Fourth Dataset (52 Features—Four Classes)—Data Augmentation—3BGRU Architecture

The model was trained on 12052 input sequences, each one composed of 120 frames, with a 50% overlap and described by the 52 features. The validation dataset was obtained as a fraction of the training data (10%). The test sequences were 989 in total, as they were not involved in the data augmentation procedure.

The mean accuracy value of the model was equal to 0.88 ± 0.01.

The results shown in Table 4 suggest that this model performed well overall, achieving high mean specificity values for all classes (ranging from 0.95 ± 0.01 to 0.99 ± 0.01), indicating a low rate of FP. The mean sensitivity values of Class 2 and Class 4 were relatively low, at 0.85 ± 0.02 and 0.86 ± 0.04, respectively, suggesting that the model had more difficulty in correctly identifying TP for these classes. However, the mean sensitivity values of Class 1 were high, at 0.93 ± 0.02, indicating that the model was able to accurately identify TP for this class. The mean precision values ranged from 0.79 ± 0.09 for Class 4 to 0.92 ± 0.02 for Class 2 and Class 3, suggesting that the model had some difficulty in distinguishing between TP and FP for Class 4.

**Figure 11:**
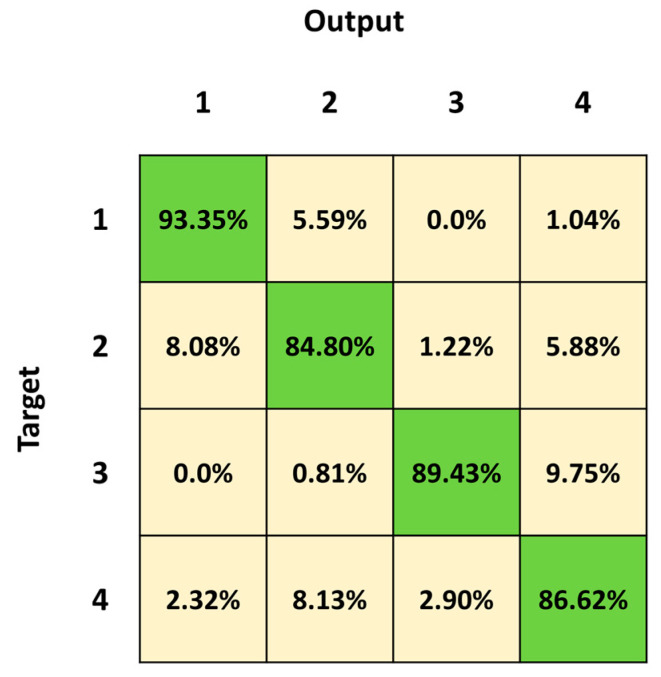
Mean confusion matrix obtained from the 30 3BGRU network simulations on the *fourth dataset*.

Figure 11 shows the mean confusion matrix computed over the 30 network simulations. The major misclassifications occur in Class 2 and Class 4, with a percentage of correct classifications of 84.80% and 86.62%, respectively. Class 2 is mainly confused with Class 1 (8.08%), whereas Class 1 is exchanged with Class 2 (5.59%). The best identified class is Class 1 (standing posture), followed by Class 3 (lying posture).

**Figure 12:**
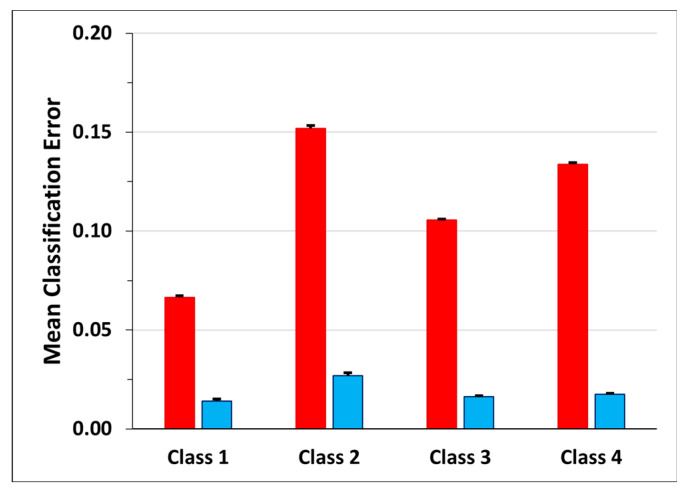
Mean classification error for each class. The red bars represent the percentage of FN for each class, while the blue bars represent the percentage of FN that corresponds to frames that were previously considered transitions and now are labeled as pertaining to the class.

Observing the mean classification errors of Figure 12 (red bars), the smallest mean error occurs for Class 1 (0.07 ± 0.09), followed by Class 3 (0.11 ± 0.04) and Class 4 (0.13 ± 0.09). Class 2 shows the highest value (0.15 ± 0.13) of mean classification error together with that of mean transition frames error ratio (blue bar, 0.025 ± 0.14).

### 3.6. Performance Comparisons between the Two 5-Classes Identification Models

A *t*-test was run on the mean accuracy results to assess the differences in performance between the 3BGRU architecture with eight features and the same architecture with 52 features when classifying five classes (t(58) = 4.66, *p* < 0.001). Notably, the 3BGRU architecture performed better when eight features were used.

**Figure 13:**
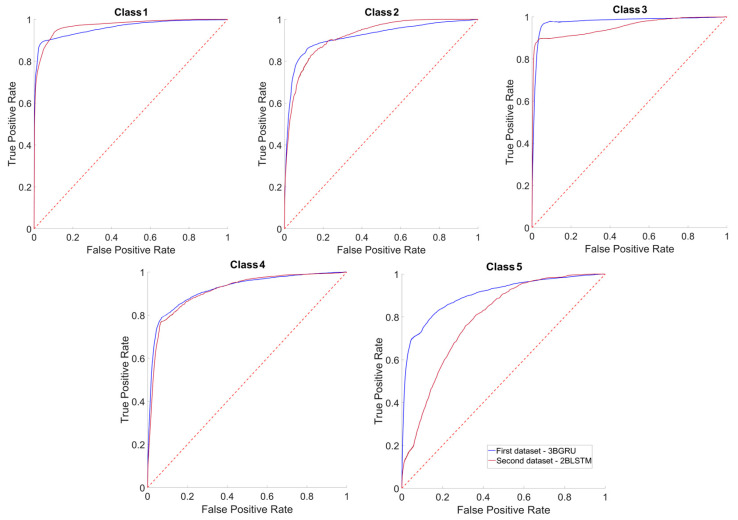
Average ROC curves of each class considered. For each graph, the curve referred to the data of the *first dataset* 3BGRU (blue line) is superimposed on that of the *second dataset* 3BGRU (red line). The confidence intervals of each average ROC curve are not shown, for purposes of clarity.

Figure 13 depicts the average ROC curves of the two models separately for each one of the five considered classes. The mean values of the Area Under the Curve (AUC) were greater than 0.90, i.e., not far from 1, for both models and for all classes except for Class 5 with the 3BGRU—*second dataset*, which was equal to 0.79 ± 0.02. This mean value was statistically lower than that of the same class obtained for the 3BGRU—*first dataset* (0.90 ± 0.01; t(58) = 26.83, *p* << 0.001). A similar behavior was found for Class 3 (0.95 ± 0.01 (3BGRU—*second dataset*) and 0.98 ± 0.01 (3BGRU—*first dataset*), t(58) = 9.80, *p* << 0.001), whereas the mean AUC of Class 1 was higher for the 3BGRU—*second dataset* against that of the same architecture with the *first dataset* (0.97 ± 0.00 and 0.96 ± 0.01, respectively t(58) = 6.41, *p* << 0.001).

**Figure 14:**
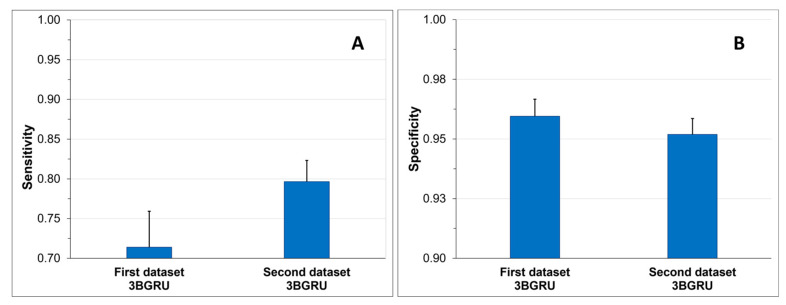
Sensitivity (**A**) and specificity (**B**) mean results + SD.

To assess the ability of the two models to correctly identify Class 4 sequences, TP and TN rates, also known as sensitivity (Figure 14A) and specificity (Figure 14B), were compared. The 3BGRU architecture with eight features exhibited a significantly lower mean sensitivity (0.71 ± 0.04) compared to the same architecture with 52 features (t(58) = 8.67, *p* < 0.001), while it showed a higher sensitivity towards the identification of TN (0.96 ± 0.01 and 0.95 ± 0.01, *t*-test: (t(58) = 4.31, *p* < 0.001).

### 3.7. Performance Comparisons between the Three Four-Classes Identification Models

By comparing the mean accuracy results of the model classifying four classes, we found statistically significant differences (one-way ANOVA, F(2,87) = 65.76, *p* < 0.001). The Bonferroni post-hoc test showed that the mean accuracy was significantly different among the models (*p* < 0.001, for all comparisons). The best performance was obtained by the 3BGRU architecture with 52 features trained with the balanced dataset obtained after applying the data augmentation technique (*fourth dataset*). When no data augmentation was performed, the GRU architecture remained the best, when compared with the 2BLSTM.

**Figure 15:**
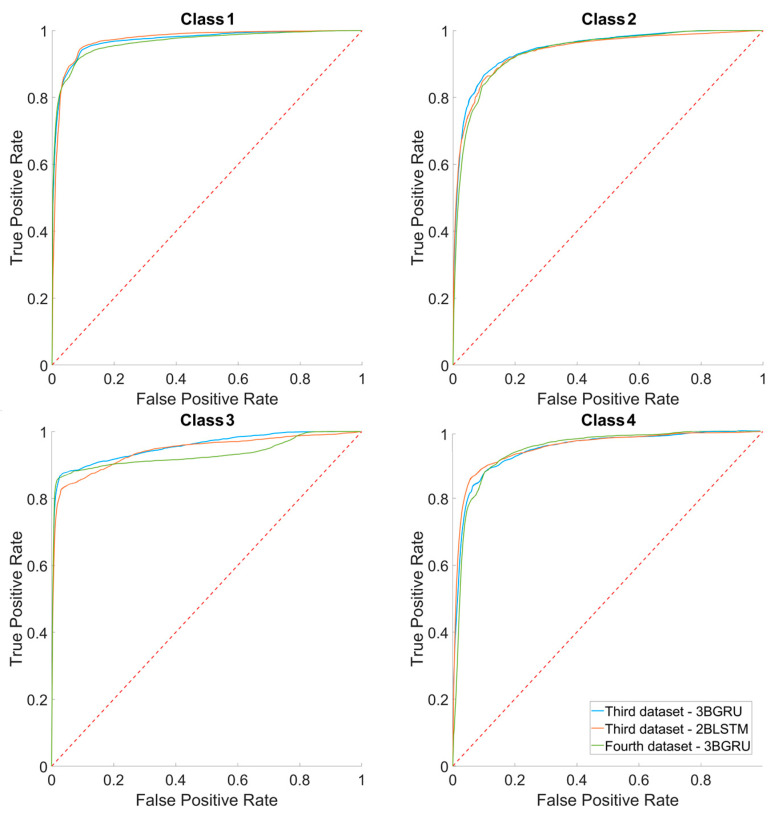
The average ROC curves over the 30 simulations for each class, respectively, for *third dataset* 3BGRU, *third dataset* 2BLSTM and *fourth dataset* 3BGRU. The confidence intervals of each ROC curve are not shown, for purposes of clarity.

Figure 15 shows the superimposed average ROC curves of the three models classifying four classes. For all models, the AUC mean values for Class 1 were close to 1 and higher than those of the other classes (0.97 ± 0.01, 0.97 ± 0.01 and 0.97 ± 0.00 for the 3BGRU—*third dataset*, 2BLSTM—*third dataset* and 3BGRU—*fourth dataset*, respectively). The AUC mean values for Class 3 were significantly different among the models (one-way ANOVA, F(2,87) = 20.88, *p <*< 0.001); in particular, that of 3BGRU—*fourth dataset* (0.93 ± 0.01) was significantly lower with respect to that of 3BGRU—*third dataset* (0.95 ± 0.01) and 2BLSTM—*third dataset* (0.94 ± 0.02), (*p <*< 0.001).

**Figure 16:**
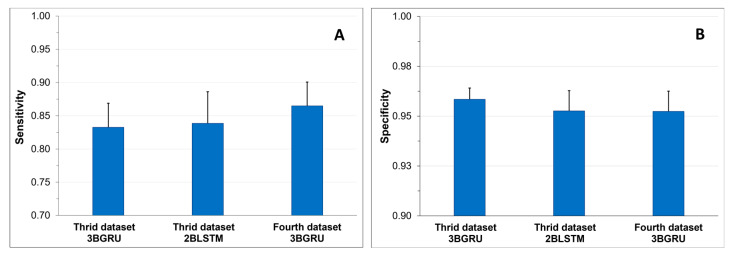
Mean sensitivity results + SD (**A**) and mean specificity results + SD (**B**).

To assess the ability of the three models to correctly identify Class 4 sequences, the sensitivity (Figure 16A) and specificity (Figure 16B) were compared among the models. Statistically significant differences were found among the mean specificity results of the models (one-way ANOVA, F(2,87) = 4.57, *p* < 0.015). The Bonferroni post-hoc test demonstrated that the mean specificity of the 3BGRU architecture with 52 features (*third dataset*) was significantly higher with respect to that of each of the other two models (*p* < 0.037, for all comparisons). The data augmentation technique, applied to increase the numerosity of Class 3 and Class 4 (*fourth dataset*), reduced the mean specificity of the 3BGRU architecture in the identification of Class 4 (Figure 16A). The same statistical analysis was performed on the mean sensitivity results to investigate the capability of the different models to produce TP of Class 4 (Figure 16A). The best mean sensitivity was that of the 3BGRU architecture with data augmentation (*fourth dataset*, 0.86 ± 0.04), which was statistically different from that of the other two models (one-way ANOVA, F(2,87) = 5.51, *p* < 0.007; Bonferroni post-hoc test, *p* < 0.038, for all comparisons).

## 4. Discussion

Several RNN network model solutions have been described and tested in this paper. Differently from our previous studies, which aimed at classifying individual video frames, all models were here sequence-to-last, i.e., producing a single classification for each presented sequence of frames.

In order to analyze their performance statistically, all models were trained 30 times, for 60 epochs each. Training and testing were performed on *four datasets* that were built over the same acquisitions yet considering different numbers and types of features and/or classes. All the training datasets considered data from the same subset of ten subjects, and the test datasets were built from the remaining two acquired subjects.

We first trained and tested the 3BGRU (three Bidirectional GRU layers, alternated by four dropout layers and three fully connected layer), with the *first dataset* (Table 1) composed of eight features and labeled with five classes (Class 1: standing posture, Class 2: sitting posture, Class 3: lying posture, Class 4: dangerous-sitting posture and Class 5: transition posture). The 3BGRU model achieved a mean accuracy of 82%, while the 2BLSTM2D model proposed in our previous work obtained a mean accuracy of 85% [30]. Looking to improve these results and inspired by the work of Wang et al. [33], we then built a new dataset (*second dataset*, Table 2) described by a new set of features (the 51 joint egocentric coordinates plus the Euclidean distance of the subject from the camera). We trained and tested the 3BGRU model with the *second dataset* (Table 1), reaching a mean accuracy value of 81%, very close to the one obtained previously. However, examining in detail Figure 5 and Figure 6, it is possible to note that the 3BGRU model trained with the *second dataset* compared to the model trained with the *first dataset* better identified Class 1 and Class 4 (from 88.78% to 95.52% and from 71.84% to 80.24%, respectively); the performance over Class 2 remained almost unchanged (respectively, 82.24% and 80.10%), while Class 3 and especially Class 5 worsened significantly (respectively, from 96.92% to 87.24% and from 62.69% to 19.05%). Considering the behavior for Class 5, we studied a new data labelling in which the transition between two consecutive postures was identified with the posture following the transition. The 3BGRU model was then trained and tested on such a *third dataset* (Table 2). In this case, a mean accuracy of 87% was achieved on the test database. This turned out to be the best result achieved so far in terms of accuracy. The comparison between Figure 6 and Figure 7 confirms the improved performances of the 3BGRU model trained with *third dataset*. While Class 1 is identified as slightly worse (respectively, from 95.52% to 91.26%) and the performance with Class 3 is unchanged (from 87.93% to 87.80), Class 2 and Class 4 are now better recognized (from 80.10% to 85.04% and from 80.24% to 83.13%, respectively). These results concerning Class 4 are very important for the purpose of the home monitoring system, since it is designed to recognize dangerous situations (Class 4) immediately after these have occurred, and not while they are occurring (transition phase between two postures).

To validate the performance of the 3BGRU model on the *third dataset*, we compared it with the 2BLSTM architecture proposed in a previous work [30] yet configured for sequence-to-last classification and the new numbers of inputs and outputs, trained and tested with the same dataset for 30 simulations of 60 epochs each. In these conditions, the 2BLSTM model reached a mean accuracy of 85%, significantly lower than that of the 3BGRU model. Moreover, observing in detail the performance of the individual classes, the misclassification error between Class 3 and Class 2 in the 3BGRU model decreased (Figure 7 and Figure 9). This was confirmed by the mean sensibility values for Class 3, which was 0.87 ± 0.02 for the 3BGRU model and only 0.80 ± 0.07 for the 2BLSTM one, and for Class 2, which was 0.85 ± 0.03 for the 3BGRU model compared to 0.80 ± 0.03 (Table 4).

In addition, to further improve the generalization abilities of the 3BGRU model, noting that in the training database the number of sequences pertaining to the four classes were highly unbalanced (Figure 3, blue bars), we decided to apply a data augmentation technique. We therefore implemented a data augmentation method based on adding Gaussian noise only to the training sequences identified with Class 3 and Class 4, i.e., those most strongly underrepresented in the dataset (Figure 3, blue bars). With the data augmentation solution, we obtained a new dataset, the *fourth dataset* (Table 2). The 3BGRU model trained and tested with the *fourth dataset* achieved a mean accuracy of 88%, which was significantly higher with respect to that of the model trained without the augmented data, i.e., achieved a close mean accuracy value of, respectively, 88% and 87%. Regarding the 3BGRU model trained with the *fourth dataset*, (Table 4) relative to the dangerous-sitting posture (Class 4), the sensitivity increased from a value of 0.83 ± 0.04 to a value of 0.86 ± 0.04, yet the precision decreased from 0.81 ± 0.02 to 0.79 ± 0.09.

This was also confirmed by the confusion matrices in Figure 7 and Figure 11. In particular, the percentage of true positives related to Class 4 increased from 83.13% to 86.62%, and the percentage of false negatives related to Class 4 accordingly decreased from a percentage equal to 16.87 to 13.38 (2.90%, 8.72% and 5.23% with the third and 2.32%, 8.13% and 2.90% with the fourth, respectively, for Class 1, Class 2 and Class 3).

In sum, we developed a new deep learning model based on GRU layers (3BGRU), for investigating a different RNN solution with about half the number of hyperparameters with respect to the previously proposed network (2BLSTM2D), based on LSTM layers (about 220 k free parameters vs. 460 k) [30]. A lower number of hyperparameters could represent a helpful condition in our context, given the limited amount of data in our custom dataset. In addition, the 3BGRU model, compared to the 2BLSTM2D, has an extra layer (3 BGRU vs. 2 LSTM layers), which could improve the model’s discriminative capacity, increasing the classification performance. With this approach, we also adopted a sequence-to-last classification, making a single prediction of the subject’s posture for each input data sequence, corresponding to four seconds of recordings (120 frames). Finally, the 3BGRU model, which was demonstrated to be better for our purpose, was the one trained with the augmented data, since it improved the identification of the dangerous-sitting posture (Class 4), yet its specificity slightly decreased compared to the training without augmented data.

As a final remark, the mean accuracy achieved by our best model was generally lower than those found in the reference literature, which is around 90% [18,45,46]. Generally, these accuracy results are obtained by analyzing public datasets containing data that are either not representative of the everyday life conditions needed for our purpose, or acquired with experimental setups in which the subject is static and facing the camera (optimal condition for the Kinect V2 video recordings). Our dataset was tailored to the home monitoring system under development in our Lab, and to this aim it was based on postures acquired during everyday life scenarios in which the subjects were free to be frontal to or turned sideways with respect to the Kinect camera as in natural living conditions. Unfortunately, this realistic approach increased the amount of noise in our dataset, making the classification process more complex and likely reducing the accuracy of the implemented models.

### Limitations of the Proposed Work

The results of this paper can be helpful for Ambient Assisted Living researchers using deep learning to identify different postures during daily activities. However, there is an important limitation to this study: the small number of sequences used for model training. Collecting more data may increase the variety of the training set, thus improving the generalization ability of the model. A broader range of examples to learn from may allow the model to better capture the complex patterns present in the data and may improve its overall performance. Moreover, a large training dataset may offer the possibility to implement other deep learning models, i.e., transformer [47,48] or a combination of Convolutional Neural Networks (CNN) and GRU. This approach could help identify key features and patterns in the data that may be difficult to detect using traditional feature selection methods, thereby providing a more nuanced and accurate understanding of the underlying processes at play.

## Figures and Tables

**Table 1:** Numerosity of sequences (120 frames each) for each class, split into train and test conditions for the *First* and *Second dataset*, separately.

	*First Dataset*		*Second Dataset*	
Classes	Train	Test	Train	Test
Class 1	1601	225	2305	270
Class 2	2864	385	3172	374
Class 3	1193	230	946	117
Class 4	1559	208	1408	164
Class 5	910	128	680	64
***Total***	***7948***	***1176***	***8511***	***989***

**Table 2:** Numerosity of sequences (120 frames each) for each class, split into train and test conditions for the *Third* and *Fourth dataset*, separately.

	*Third Dataset*		*Fourth Dataset*	
Classes	Train	Test	Train	Test
Class 1	2516	286	2516	286
Class 2	3470	408	3470	408
Class 3	1016	123	3048	123
Class 4	1509	172	3018	172
***Total***	***8511***	***989***	***12,052***	***989***

**Table 3:** Specificity, sensitivity and precision mean value ± SD results, referring to the 3BGRU architecture with eight features and five classes (*first dataset*, first five rows) and with 52 features and five classes (*second dataset*, last five rows).

	Classes	Specificity	Sensitivity (Recall)	Precision
**First dataset** **(eight features)** **3BGRU**	**Class 1**	0.97 ± 0.01	0.88 ± 0.02	0.87 ± 0.03
	**Class 2**	0.92 ± 0.01	0.82 ± 0.01	0.83 ± 0.02
	**Class 3**	0.95 ± 0.01	0.96 ± 0.01	0.82 ± 0.02
	**Class 4**	0.96 ± 0.01	0.71 ± 0.04	0.79 ± 0.02
	**Class 5**	0.97 ± 0.01	0.62 ± 0.05	0.73 ± 0.03
**Second dataset** **(52 features)** **3BGRU**	**Class 1**	0.89 ± 0.01	0.95 ± 0.01	0.77 ± 0.02
	**Class 2**	0.93 ± 0.01	0.79 ± 0.02	0.88 ± 0.02
	**Class 3**	0.98 ± 0.01	0.87 ± 0.02	0.88 ± 0.02
	**Class 4**	0.95 ± 0.01	0.80 ± 0.03	0.77 ± 0.02
	**Class 5**	0.99 ± 0.01	0.19 ± 0.07	0.47 ± 0.09

**Table 4:** Specificity, sensitivity and precision mean value ± SD results, referring to the 3BGRU architecture with the *third dataset* (52 features and four classes), to the 2BLSTM architecture with the *third dataset* (52 features and four classes), and the 3BGRU architecture over the *fourth dataset* (52 features and four classes with data augmentation).

	Classes	Specificity	Sensitivity (Recall)	Precision
**Third dataset** **(52 features)** **3BGRU**	**Class 1**	0.94 ± 0.01	0.91 ± 0.03	0.86 ± 0.03
	**Class 2**	0.93 ± 0.02	0.85 ± 0.03	0.90 ± 0.02
	**Class 3**	0.98 ± 0.00	0.87 ± 0.02	0.88 ± 0.02
	**Class 4**	0.96 ± 0.01	0.83 ± 0.04	0.81 ± 0.02
**Third dataset** **(52 features)** **2BLSTM**	**Class 1**	0.92 ± 0.02	0.93 ± 0.02	0.82 ± 0.04
	**Class 2**	0.93 ± 0.02	0.80 ± 0.03	0.89 ± 0.03
	**Class 3**	0.98 ± 0.01	0.80 ± 0.07	0.87 ± 0.04
	**Class 4**	0.95 ± 0.01	0.84 ± 0.05	0.79 ± 0.03
**Fourth dataset** **(52 features)** **3BGRU**	**Class 1**	0.95 ± 0.01	0.93 ± 0.02	0.88 ± 0.02
	**Class 2**	0.95 ± 0.02	0.85 ± 0.02	0.92 ± 0.02
	**Class 3**	0.99 ± 0.01	0.89 ± 0.02	0.92 ± 0.02
	**Class 4**	0.95 ± 0.01	0.86 ± 0.04	0.79 ± 0.09
